# Human Herpesvirus-8 Infection Leads to Expansion of the Preimmune/Natural Effector B Cell Compartment

**DOI:** 10.1371/journal.pone.0015029

**Published:** 2010-11-29

**Authors:** Silvia Della Bella, Adriano Taddeo, Elena Colombo, Lucia Brambilla, Monica Bellinvia, Fabrizio Pregliasco, Monica Cappelletti, Maria Luisa Calabrò, Maria Luisa Villa

**Affiliations:** 1 Department of Biomedical Sciences and Technologies, University of Milan, Milan, Italy; 2 Institute of Dermatological Sciences, Fondazione IRCCS Ospedale Maggiore Policlinico, Mangiagalli e Regina Elena, Milan, Italy; 3 Department of Public Health, Microbiology and Virology, University of Milan, Milan, Italy; 4 Immunology and Diagnostic Molecular Oncology, Istituto Oncologico Veneto, IOV-IRCCS, Padova, Italy; University of Rochester, United States of America

## Abstract

**Background:**

Human herpesvirus-8 (HHV-8) is the etiological agent of Kaposi's sarcoma (KS) and of some lymphoproliferative disorders of B cells. Most malignancies develop after long-lasting viral dormancy, and a preventing role for both humoral and cellular immune control is suggested by the high frequency of these pathologies in immunosuppressed patients. B cells, macrophages and dendritic cells of peripheral lymphoid organs and blood represent the major reservoir of HHV-8. Due to the dual role of B cells in HHV-8 infection, both as virus reservoir and as agents of humoral immune control, we analyzed the subset distribution and the functional state of peripheral blood B cells in HHV-8-infected individuals with and without cKS.

**Methodology/Principal Findings:**

Circulating B cells and their subsets were analyzed by 6-color flow cytometry in the following groups: 1- patients HHV-8 positive with classic KS (cKS) (n = 47); 2- subjects HHV-8 positive and cKS negative (HSP) (n = 10); 3- healthy controls, HHV-8 negative and cKS negative (HC) (n = 43). The number of B cells belonging to the preimmune/natural effector compartment, including transitional, pre-naïve, naïve and MZ-like subsets, was significantly higher among HHV-8 positive subjects, with or without cKS, while was comparable to healthy controls in the antigen-experienced T-cell dependent compartment. The increased number of preimmune/natural effector B cells was associated with increased resistance to spontaneous apoptosis, while it did not correlate with HHV-8 viral load.

**Conclusions/Significance:**

Our results indicate that long-lasting HHV-8 infection promotes an imbalance in peripheral B cell subsets, perturbing the equilibrium between earlier and later steps of maturation and activation processes. This observation may broaden our understanding of the complex interplay between viral and immune factors leading HHV-8-infected individuals to develop HHV-8-associated malignancies.

## Introduction

Human herpesvirus 8 (HHV-8) is a gammaherpesvirus etiologically linked to several malignancies such as Kaposi's Sarcoma (KS), primary effusion lymphoma (PEL), and the plasmablastic form of multicentric Castleman's disease (MCD) [1]–[3]. After clearance of the initial infection, HHV-8 establishes latent infection in multiple cell types [2], [4]–[7], and it is usually detectable in malignant cells of HHV-8-related tumors. KS, a highly vascularized tumor that manifests as a multifocal angioproliferative disease of the skin and mucosa, is the most frequent HHV-8 associated malignancy. Central to KS pathogenesis is a hyperproliferation of spindle-shaped cells, that are thought to be of endothelial origin and assume the characteristic spindle shape upon infection with HHV-8. According to the multifocal nature of KS, we recently demonstrated that circulating endothelial progenitor cells cultured from the peripheral blood of cKS patients are infected by HHV-8, support viral productive replication and may therefore represent putative precursors of KS spindle cells [8]. In KS patients, B cells of peripheral lymphoid organs and blood represent a major virus reservoir endowed with the ability to sustain the lytic reactivation and dissemination of the virus [9]–[11]. Lymphoproliferative disorders of B cell origin, including PEL and MCD can occur concomitantly with KS, confirming the existence of a deep pathogenetic linkage between these malignancies [12].

The persistence of HHV-8 infection is the result of a delicate balance between immune control, viral latency, viral reactivation and persistent replication. Only a minor proportion of infected hosts eventually develop malignancies due to immune breakdown. Indeed, despite the high prevalence of HHV-8 infection (24.1% among Italian population) [13], the majority of HHV-8-positive subjects do not develop clinically evident malignancies. It is not currently understood which components of the immune responses are essential for controlling the progression from asymptomatic HHV-8 infection to development of KS. The existing data suggest that both humoral and cellular immune responses are needed; in particular the humoral response may be of vital importance in conditions of transitory or permanent reduced T cell function [14]. Taken togheter, these observations emphasize the central and conflicting role of B cells both in the maintenance as well as in the immune control of HHV-8 infection.

Peripheral B cells are morphologically homogenous, but their surface phenotypes, anatomic localization, and functional properties reveal a great level of complexity, which may be disrupted by viral infection and inflammation. Immature B cells exit the bone marrow and in the periphery they mature via transitional stages into either mature naïve or marginal zone (MZ) B cells, which have mixed attributes of naïve and memory B cells [15]. Because transitional B cells and MZ B cells may respond to T cell-independent antigens, they are indicated as natural effector cells [16]. Together with mature naïve B cells they constitute the preimmune/natural effector compartment. Upon activation by T helper-dependent antigens, mature naïve B cells progress into switched and IgM activated/memory B cells. These cells represent the compartment of antigen-experienced cells [17]. A complex regulation of several negative and positive selection checkpoints usually controls peripheral B cell development [18]. At present, abnormalities in one or more B cell subsets have been found across a broad range of infectious and non-infectious diseases [19]–[22]. HHV-8 is a good potential candidate as B cell disregulating agent, because it is able to influence the fate and function of B cells through a number of viral proteins that affect B cell development, allow infected B cells to escape from the control of the immune system, and trigger clonal B cell proliferation by affecting B cell cycle check-points and by mimicking cell signals that control cell proliferation [2].

In order to better understand the role of B cells in chronic HHV-8 infection and associated malignancies, in this study we fully characterized peripheral B cells in HHV-8 positive patients with or without KS compared with healthy controls. To avoid the interference of confounding factors related to iatrogenic or AIDS-related KS, we studied patients affected by the classic, Mediterranean variant of the disease [1]. We showed that cKS patients have increased number of circulating B cells, characterized by an expansion of transitional, pre-naïve, naïve and MZ-like cells, all of which showed an increased resistance to apoptosis. Antigen-experienced memory B cell subsets appeared unaffected. No signs of increased B cell activation nor increased incidence of monoclonal B cell expansions were observed. We observed changes in B cell subsets similar to those found in cKS in the group of subjects with chronic HHV-8 infection without KS, suggesting that HHV-8 infection per se may be responsible for perturbation of B cell homeostasis in KS patients.

Abnormalities in B cell subsets, with altered equilibrium between earlier and later steps of maturation and activation processes, have never been described in HHV-8 infected patients; we suggest that these observations may open the way to a better understanding of the role of inflammation and immunity in the control of gammaherpesvirus infections and their neoplastic outcomes.

## Results

### Total B cells and their transitional, pre-naïve, naïve and MZ-like subsets are expanded in cKS patients

**Figure 1:**
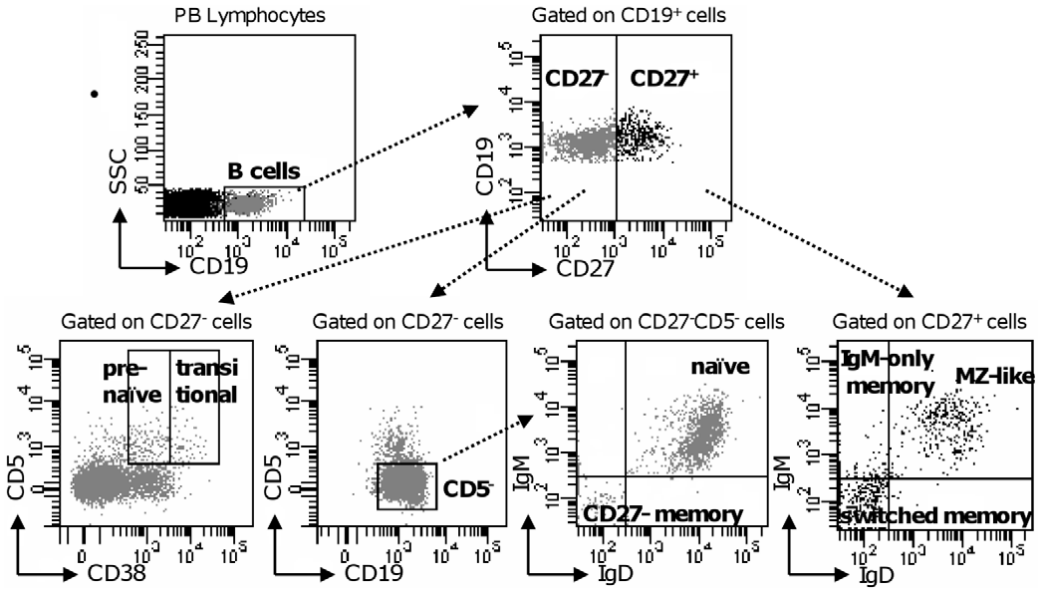
Identification of B cell subsets in the peripheral blood. Gating strategy used to identify circulating B lymphocytes and their subsets by flow cytometry. Peripheral blood samples from cKS patients, HSP subjects and HCs were stained as described in Methods. B cells were identified as CD19^+^ cells and further gated based on their CD27 surface expression. Gated on CD27^−^ B cells, transitional and pre-naïve B lymphocytes were identified as CD5^+^CD38^hi^ and CD5^+^CD38^int^ cells, respectively. Gated on CD27^−^CD5^−^ B cells, naïve and CD27^−^ memory B lymphocytes were identified as IgD^+^IgM^+^ and IgD^−^IgM^−^ cells, respectively. Gated on CD27^+^ B cells, switched memory, IgM-only memory and MZ-like B lymphocytes were identified as IgD^−^IgM^−^, IgD^−^IgM^+^ and IgD^lo^IgM^+^ cells, respectively. A representative flow cytometric analysis is shown.

We examined the frequency and number of B cells and their subsets in the peripheral blood of 47 cKS patients compared with 43 HCs, by using 6-color flow cytometry. As shown in Figure 1, B cells were identified in PB samples as CD19^+^. Gated on B cells, CD27^−^ and CD27^+^ B cells were defined. Within CD27^−^ cells, transitional B cells were identified as CD5^+^CD38^hi^ cells, pre-naïve B cells as CD5^+^CD38^int^, unswitched naïve B cells as CD5^−^IgD^+^IgM^+^, CD27^−^ memory B cells as CD5^−^IgD^−^IgM^−^ cells [23], [24]. Within CD27^+^ cells, switched memory B cells were identified as IgD^−^IgM^−^ cells, IgM-only memory B cells as IgD^−^IgM^+^ and MZ-like B cells as IgD^lo^IgM^+^ cells [25]–[27].

**Figure 2:**
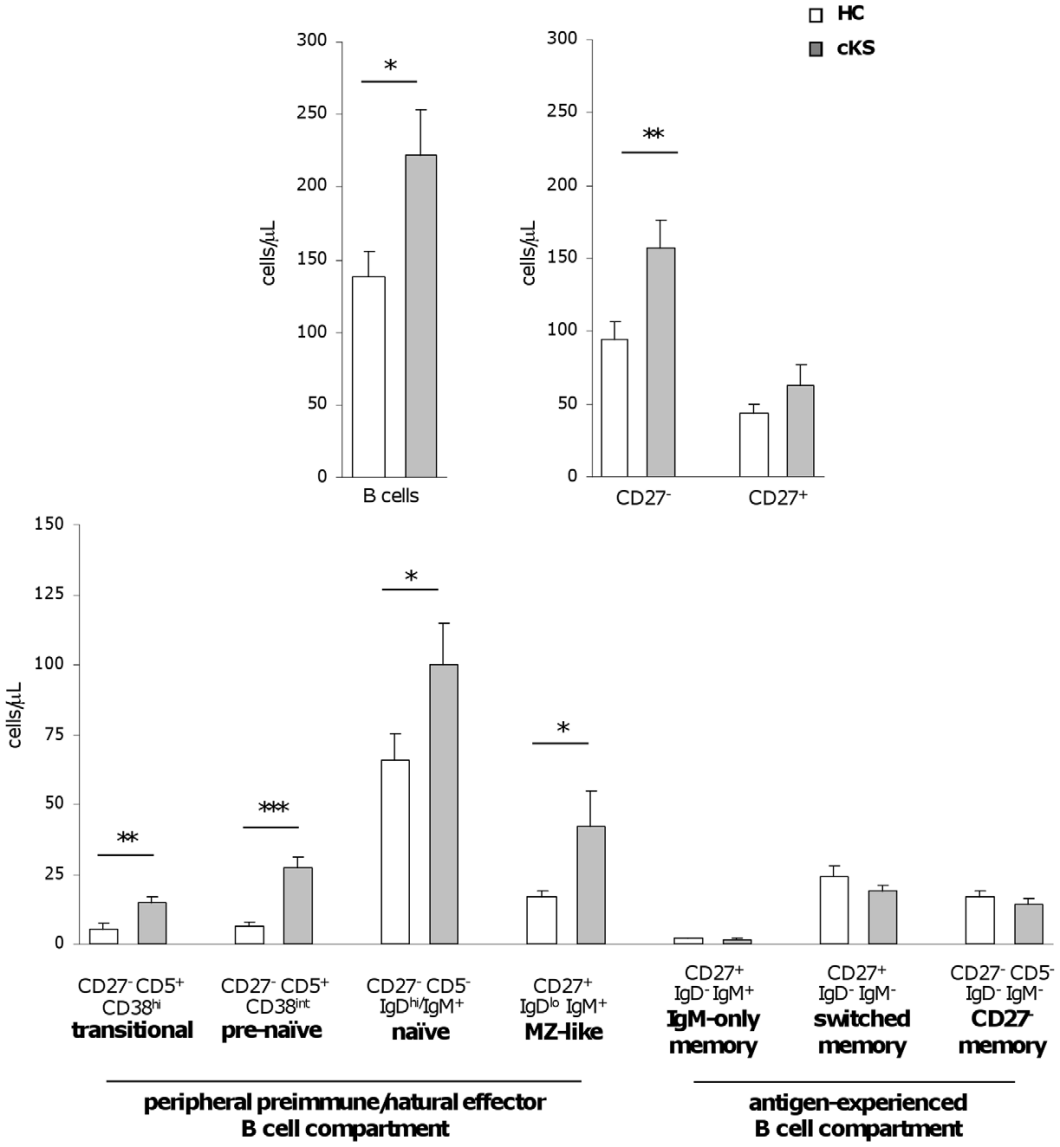
B cells and their non-memory subsets are increased in patients with cKS. The number of total B cells and CD27^−^ B cells was significantly higher in cKS patients (grey bars) than HCs (white bars). All the subsets composing the preimmune/natural effector compartment, namely transitional, pre-naïve, naïve and MZ-like B lymphocytes, were increased in cKS patients, while the subsets composing the antigen-experienced T cell-dependent compartment, namely IgM-only, switched and CD27^−^ memory B cells, were unaffected. Data shown as mean ± SE. *P*-values calculated using the Student *t* test for independent samples. *P<.05; **P<.01; ***P<.001.

The frequency of B cells within lymphocytes in aged HCs was 6.7% ±0.5, as expected [28], [29], and it was significantly higher in cKS patients (10.9% ±1.1; *P*<0.001). As shown in Figure 2, the absolute number of B lymphocytes was accordingly expanded in cKS patients than in HCs (*P* = 0.02). To more precisely define the phenotype of the expanded populations we examined the distribution of B cells along the various steps of peripheral B cell maturation and differentiation. The frequency of CD27^−^ B lymphocytes was significantly higher in cKS than HCs (73.6% ±2.1 vs 67.1% ±2.6; *P*<0.05). This resulted in a significant increase of the absolute number of CD27^−^ but not CD27^+^ B cells (Figure 2). As shown in the same figure, the absolute number of transitional, pre-naïve, naïve and MZ-like B cells, all composing the peripheral preimmune compartment, resulted significantly higher in cKS patients than HCs (*P* = 0.002, *P*<0.0001, *P*<0.05 and *P*<0.05, respectively). On the contrary, the size of memory B subsets, including IgM-only, isotype-switched and CD27^−^ memory B cells, did not change between cKS patients and healthy controls. No correlation was observed between changes in B cell subset distribution and the clinical stage or evolution of KS.

### The expanded B cell subsets from cKS patients show increased resistance to spontaneous apoptosis and unaffected in vivo turnover

To investigate the mechanisms possibly underlying the expansion of distinct B cell subsets in cKS patients, we examined two cell parameters that can impact on the size of cell populations and that can be measured in the periphery: cell apoptosis and cell turnover.

**Figure 3:**
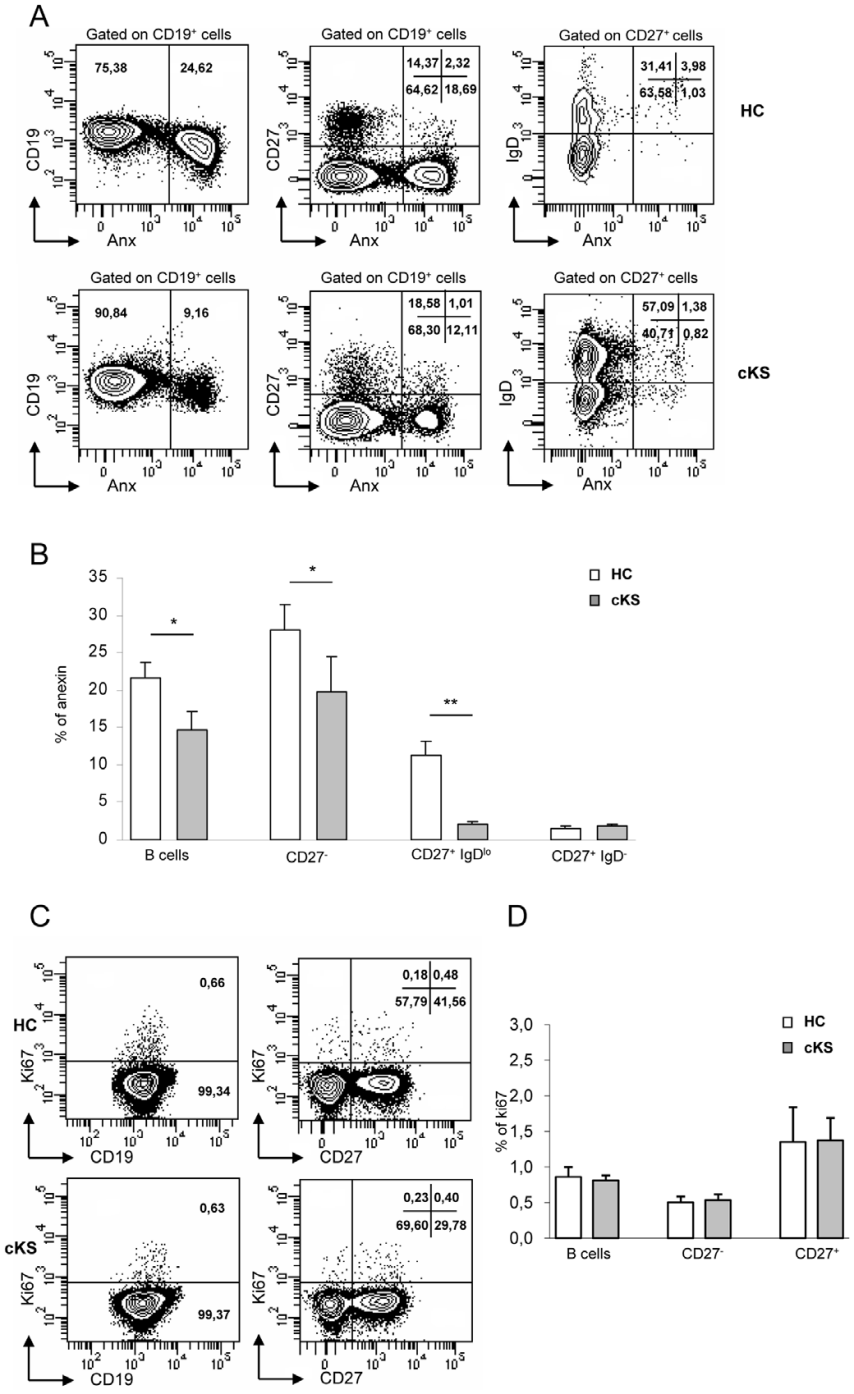
B cells from cKS patients show increased resistance to spontaneous apoptosis and unaffected *in vivo* turnover. Apoptotic cells were detected by annexin V binding after 24 h culture in unstimulated conditions. *A*, representative flow cytometric analysis showing annexin V binding gated on total B cells, CD27^−^, MZ-like (CD27^+^IgD^lo^) and switched memory (CD27^+^IgD^−^) B cells, as indicated; comparison between one HC (upper row) and one cKS patient (lower row). B, B cells from cKS patients (grey bars) showed significantly lower annexinV binding than B cells from HCs (white bars); this lower annexin V binding was evident on CD27^−^ and MZ-like (CD27^+^IgD^lo^) B cells, roughly composing the preimmune/natural effector compartment, but not on antigen-experienced switched memory (CD27^+^IgD^−^) B cells. Data shown as mean ± SE. *C, In vivo* cell turnover was estimated by Ki67 staining of freshly isolated PBMCs. Representative flow cytofluorimetric analysis showing Ki67 binding gated on total B cells, CD27^−^ and CD27^+^ B cells, as indicated; comparison between one HC (upper row) and one cKS patient (lower row). *D*, The proportion of Ki67^+^ cells within total B cells, CD27^−^ and CD27^+^ B cells did not differ between cKS patients (grey bars) and HCs (white bars). Data shown as mean ± SE. *P*-values calculated using the Student *t* test for independent samples.*P<.05; **P<.01.

The level of spontaneous apoptosis of cultured B cells, as detected by annexin V binding (Figure 3*A*), was significantly lower in cKS patients than in healthy donors (*P*<0.05) (Figure 3*B*). As shown in the same figure, a significantly lower apoptosis of cKS cells was observed in CD27^−^ cells (*P*<0.02), mainly composed of transitional, pre-naïve and naïve B cells, as well as in CD27^+^IgD^lo^ cells (*P*<0.01), consisting of MZ-like B cells. Because all the above populations were expanded indeed in cKS, it may be argued that resistance to apoptosis may contribute at least in part to the perturbation of B cell homeostasis observed in our patients. Accordingly, CD27^+^IgD^−^cells that mainly consist of switched memory B cells and did not vary between patients and controls, did not show differences between groups in sensitivity to apoptosis. The analysis of cell apoptosis gated on different B cell subsets also showed that both in patients and controls the frequency of apoptotic cells was lower in CD27^−^ than CD27^+^ subsets, as expected [30].

To estimate the *in vivo* turnover of B cells, we analyzed the expression of Ki67, a nuclear antigen that identifies recently divided cells. A representative flow cytometric analysis is shown in Figure 3*C*. The proportion of Ki67^+^ cells was higher within CD27^+^ than CD27^−^ B cells, as expected [31]. As shown in Fig. 3*D*, the percentage of Ki67^+^ cells among total B cells, as well as CD27^−^ and CD27^+^ B cells did not differ between patients and controls, making unlikely the possibility that the expansion of distinct CD27^−^ subsets and MZ-like B cells observed in our cKS patients may rely on increased spontaneous turnover.

### B cells from cKS patients have a resting phenotype

**Figure 4:**
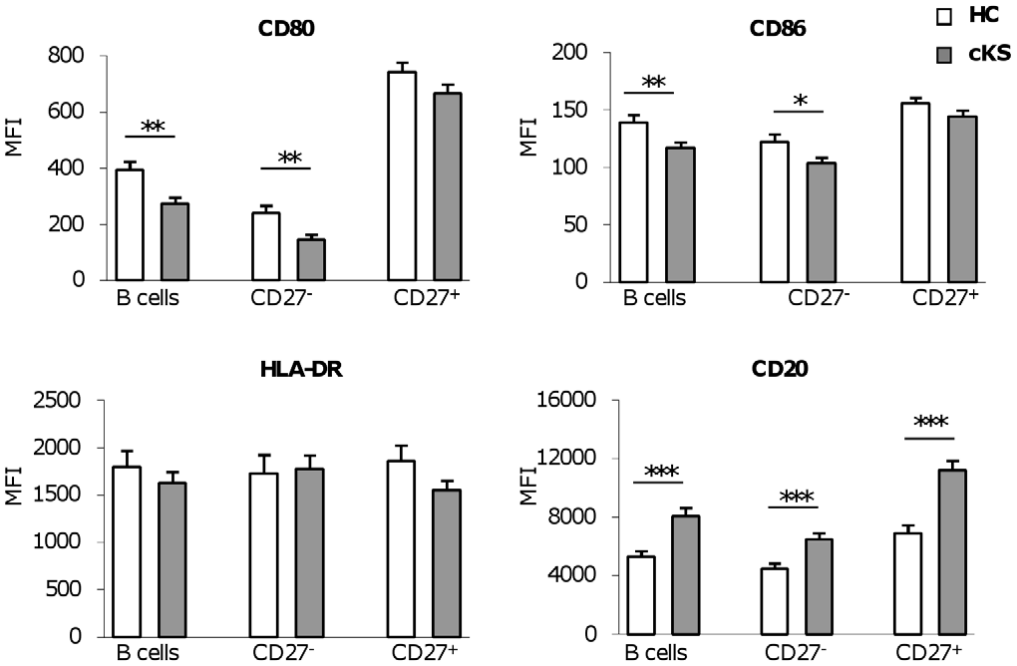
B cells from cKS patients show a low state of activation. B cells from cKS patients (grey bars) expressed lower levels of the costimulatory molecules CD80 and CD86 and higher levels of CD20 than B cells from HCs (white bars). The expression of the indicated markers on total B cells and their CD27^−^ and CD27^+^ subsets is shown. Data presented as mean ± SE of mean fluorescence intensity (MFI) values. *P*-values calculated using the Student *t* test for independent samples. *P<.05; **P<.01; ***P<.001.

We further investigated the state of activation of B cells in KS patients. Common manifestations of B cell hyperreactivity, like hypergammaglobulinemia and increased frequency of autoantibodies, were lacking (gammaglobulinemia in cKS patients and HCs: 17.3% ±0.6 and 16.7% ±0.8, respectively; positive ANA in cKS patients and HCs: 21% and 18%, respectively). We also examined the expression of activation markers that may be up-regulated on B lymphocytes upon chronic antigenic or polyclonal stimulation. In particular, we analyzed the expression of the costimulatory molecules CD80 and CD86 and of HLA-DR. As shown in Figure 4, the intensity of expression of both costimulatory molecules, expressed as mean fluorescence intensity (MFI), was significantly lower on B cells from cKS patients than HCs (*P*<0.002 and *P*<0.005 for CD80 and CD86, respectively). The lower CD80 and CD86 expression was equally evident on B cells of the expanded CD27^−^ compartment and on CD27^+^ B cells, although in this latter case the difference did not reach the statistical significance. As shown in the same Figure, also the expression of HLA-DR molecules tended to be lower on B cells from KS patients than HCs. We further analyzed B cell expression of CD20, a putative calcium channel that is down-regulated upon B cell activation and that is expressed by the majority of human B-cell lymphomas and leukemias [32], [33]. According to their immunophenotype indicative for a state of lower activation, B lymphocytes from cKS patients expressed higher surface levels of the molecule CD20 (*P*<0.001) (Figure 4). As observed for the other activation markers, the higher expression of CD20 was quite evident on CD27^−^ as well as CD27^+^ B cells (*P*<0.001 and *P*<0.0001, respectively). Similar results were obtained when the frequency of B cells expressing the activation markers, rather than MFI of their expression, was considered. No correlation was observed between the activation of B cells and the clinical stage or evolution of KS.

### The frequency of monoclonal B cell expansions is not increased in cKS patients

Because chronic HHV-8 infection may result in the development of lymphoproliferative disorders, we further characterized B cells to reveal a possible increased frequency of monoclonal B cell expansions in our cKS patients. To this aim, we analyzed the immunophenotype of B cells and evaluated the κ/λ light chain ratio in cKS patients and HCs, according to the diagnostic criteria proposed for monoclonal B lymphocytosis (MBL) [34], [35]. In particular, CLL-like MBL was defined based on the distinct CD5^bright^CD20^dim^ expression pattern on CD19^+^ B cells. Atypical CLL-like and non-CLL-like MBL were defined based on the occurrence of an unbalanced κ/λ ratio (more than 3∶1 or less than 1∶3) within CD5^+^ and CD5^−^ B cells, respectively. Following this strategy, we identified MBL in 4 of 43 HCs (9.3%; 1 CLL-like, 2 atypical CCL-like and 1 non-CLL-like MBL), as expected [34], [35]. Moreover, we identified MBL in 2 of 47 cKS patents (4.3%; 1 CLL-like and 1 non-CLL-like MBL), thus indicating that cKS patients do not have an increased incidence of monoclonal B cell expansions. κ light chain restriction was observed in 2 MBL clones (2 HCs), λ light chain restriction in 2 clones (1 HC and 1 cKS patient), while negativity for both light chains was evident in the remaining 2 cases.

### Healthy HHV-8 seropositive (HSP) controls have changes in B cells similar to those observed in cKS patients

To investigate whether the changes observed in circulating B cells from cKS patients were secondary to HHV-8 infection per se, or whether they were related to the presence of the tumor, we included in our study ten age- and sex-matched HSP controls without KS. We analyzed the number of B cells and their subsets and the state of activation of B cells in HSP subjects compared with HCs and cKS patients. As reported in Table 1, HSP controls showed changes in B cells that were similar to those observed in cKS patients, although they were evident at a lesser extent. In particular, HSP had higher number of total B cells than HCs (*P*<0.01); similarly to what we observed in cKS patients, the expansion of B cells in HSP controls was mainly restricted to the CD27^−^ population, that was markedly increased in HSP compared with HCs (*P*<0.002). As observed in cKS patients, HSP controls showed expanded transitional, pre-naïve, naïve and MZ-like B cells, while the number of IgM-only, switched and CD27^−^ memory B cells was unaffected. To further investigate whether B cells from HSP controls also showed the immunophenotypic changes observed in cKS patients indicative for a state of lower activation, we analyzed B cell expression of CD80 and CD86 and observed indeed that the intensity of expression of both costimulatory molecules, expressed as MFI, was significantly lower on B cells from HSP than HCs (*P*<0.02 and *P*<0.05 for CD80 and CD86, respectively) (Table 1). All together, these results suggest that the perturbation of B cell homeostasis occurring in KS patients is related to HHV-8 infection per se, as the presence of KS is not a condition strictly required to induce the unbalance of peripheral B cell subsets.

**Table 1:** Analysis of B cells from healthy HHV-8-seropositive (HSP) compared with HHV-8-seronegative controls (HC) and cKS patients (cKS).

			HC	cKS	HSP	p values HSP vs HC^b^	p values HSP vs cKS^b^
No.			43	47	10		
**Cells count (cells/µL)** a							
		B cells	128.53±12.85	234.41±35.28	187.19±19.26	0.0026	0.3926
		CD27^−^ B cells	85.56±9.11	172.38±24.30	140.6±17.13	0.0012	0.3845
		CD27^+^ B cells	42.97±5.79	58.54±12.21	50.37±9.12	0.1374	0.2127
	peripheral preimmune pools:						
		transitional	5.29±2.03	14.67±2.03	8.39±1.56	0.0003	0.1891
		pre-naïve	6.58±1.35	27.15±3.73	15.46±2.89	0.0023	0.2873
		naïve	65.7±9.6	98.24±14.87	88.93±12.81	0.0425	0.3132
		MZ-like	16.59±2.38	42.12±12.45	22.39±4.17	0.0499	0.4913
	antigen-experienced pools:						
		IgM-only memory	1.96±0.38	1.68±0.26	1.86±0.42	0.6289	0.2564
		switched memory	24.35±3.54	19.03±2.23	25.5±5.69	0.2993	0.1155
		CD27^−^ memory	16.95±2.15	14.28±2.17	13.11±2.43	0.3559	0.2605
**B cell activation**							
		CD80	394.28±29.15	273.24±22.94	272.50±35.86	0.0158	0.2898
		CD86	139.02±6.31	116.73±4.83	113.78±9.63	0.0499	0.4856

a Mean ± standard error.

b Mann-Whitney U test was used to detect significant differences.

### Changes in B cells from cKS patients and HSP controls do not correlate with HHV-8 load

To evaluate whether the entity of B cell alterations observed in cKS patients and HSP controls was related to HHV-8 viral load, we analyzed possible correlations between the number of B cells or their subsets and the viral gEq measured in plasma. No correlation was observed between the count of either total, CD27^−^, CD27^+^ B cells or their subsets and plasmatic HHV-8 gEq. To further investigate a possible role of B cell infection in promoting the expansion of B cells and their subsets, we selected 7 cKS patients and 6 HSP controls with B cell count either over the 75^th^ percentile or under the 25^th^ percentile calculated for each group, and determined the presence of HHV-8 gEq in their PBMCs and isolated B cell and non-B fractions. As reported in Table 2, B cells from 6 of 7 cKS patients and from 3 of 6 HSP controls were found to harbor HHV-8 DNA sequences (6–475 HHV-8 gEq/10^5^ cells). HHV-8 DNA was detected in B cells from 5 of 7 subjects with B cell expansion and from 4 of 6 subjects without expansion; moreover, the number of HHV-8 gEq did not differ between individuals with or without B cell expansions, thus indicating a lack of correlation between B cell viral infection and B cell changes. Accordingly, no correlation was observed between anti-HHV-8 antibody titres and B cell changes.

**Table 2:** HHV-8 load in isolated PBMCs, B cells and non-B cells from cKS patients and healthy HHV-8-seropositive (HSP) controls according to their peripheral blood B cells count.

Patient	Sex	Age	cKS Stage^a^	B cell count		gEq^b^/10^5^cells		
				Percentile	10^3^/mmc	PBMC	B cells	non-B cells
cKS 1	M	51	1A	>75^th^	491.2	<5	5.0	<5
cKS 2	M	69	1B	>75^th^	355.4	11.0	6.0	<5
cKS 3	M	69	2A	>75^th^	302.6	244.0	475.3	97.6
cKS 4	M	66	2A	>75^th^	277.8	<5	10.2	<5
cKS 5	M	74	1A	<25^th^	90.8	<5	15.4	<5
cKS 6	M	75	1B	<25^th^	69.4	<5	<5	<5
cKS 7	M	62	3A	<25^th^	43.9	<5	20.0	<5
HSP 1	M	66		>75^th^	297.8	<5	<5	<5
HSP 2	M	77		>75^th^	249.7	19.3	15.3	<5
HSP 3	M	59		>75^th^	244.2	<5	<5	<5
HSP 4	M	63		<25^th^	144.7	<5	11.0	<5
HSP 5	M	68		<25^th^	125.5	27.0	293.6	<5
HSP 6	M	71		<25^th^	107.3	<5	<5	<5

a cKS patients were classified according to our classification that takes into account the prevalent type of lesions, localization, clinical behaviour, evolutive pattern and presence of complications.

b gEq =  genome Equivalents.

### Changes in B cells from cKS patients do not correlate with the plasmatic levels IL-6 and IL-7

To evaluate whether the changes in B cells observed in our patients could be driven at least in part by cytokines known to affect the growth and differentiation of B lymphocytes, we measured the plasmatic levels of IL-6 and IL-7 in our samples. Detectable, low levels of IL-6 were found in 67% of the cKS patients (mean ± SE: 7.9±1.7 pg/ml) and 30% of the healthy controls (4.9±1.9 pg/ml) and did not significantly differ between groups. Also the plasmatic levels of IL-7, detected in all the samples, were similar in cKS patients and controls (8.6±1.0 and 7.1±0.7 pg/ml, respectively). No correlation was observed between the number of B cells or their subsets and the plasmatic levels of cytokines.

## Discussion

In this study we characterized circulating B cells in patients with cKS to investigate whether HHV-8, which is the etiologic agent of KS and has tropism for many cell types including B cells, may affect the number and/or the immune phenotype of these cells. To avoid the confounding effects of other concomitant viral infections or immunosuppressive agents, we included in the study only patients with cKS and observed that B cells from these patients differ in many aspects from those of matched healthy controls (HC). Moreover, to ascertain whether changes in B cells occurring in KS patients were reasonably secondary to HHV-8 infection per se, we included a group of healthy HHV-8-seropositive (HSP) controls lacking KS.

Using cellular markers that enable the classification of B cells into distinct subsets, we analyzed B cells belonging to either the preimmune/natural effector compartment or the antigen-experienced T helper-dependent compartment. Within the preimmune/natural effector compartment, transitional, pre-naïve and naïve B cells were identified as cells lacking the expression of CD27 and further defined by the expression of CD38, CD5 and/or Ig isotypes [23]. MZ-like B cells were identified as CD27^+^IgD^lo^IgM^+^ [25]; these cells are a unique population with attributes of both naïve and memory B cells [36]. Within the antigen-experienced T helper-dependent compartment, memory CD27^+^ B cells, including IgM-only and switched memory B cells, as well as a subset of CD27^−^IgD^−^ memory B cells [24], [25] were identified.

Our results indicated that patients with cKS, as well as HSP controls, have a significantly higher number of circulating B cells than HHV-8-seronegative healthy controls (HCs). We did not observe any correlation between B cell count and the clinical stage of KS in our patients. Neither we observed any correlation between B cells count and HHV-8 viraemia.

The expansion of B cells in cKS patients and HSP controls was restricted to the subsets of the preimmune/natural effector compartment. In particular, cKS patients and HSP controls showed an increase in transitional, pre-naïve, naïve and MZ-like B cells, while all antigen-experienced memory B cell subsets appeared unaffected. No signs of increased incidence of monoclonal B cell expansions were observed. B cells from cKS and HSP subjects were not activated, but rather expressed lower levels of costimulatory molecules than B cells from HCs, and this was equally evident on CD27^−^ and CD27^+^ cells. Furthermore, these cells did not show increased rate of cell proliferation in either CD27^−^ or CD27^+^ subset, as assessed by Ki67 staining.

The most important finding in this study was the increased resistance to spontaneous apoptosis that was confined to B cells of the expanded preimmune/natural effector compartment. Multiple HHV-8 gene products have been demonstrated to promote the survival of infected cells by increasing resistance to apoptosis, including latency-associated nuclear (LANA), viral Flice-inhibitory protein (v-FLIP) and the viral chemokine ligands v-CCL1 and v-CCL2 [4]. However, the low number of viral genomes detected in circulating B cells of our cKS and HSP patients, which is suggestive for a low frequency of infected cells, compared with the magnitude of their B cell changes seems to indicate that a direct infection of circulating B lymphocytes is not required to induce the observed B cell changes. This seems also confirmed by the lack of correlation that we observed between B cell alterations and B cell HHV-8 viral load.

Indirect mechanisms should be, therefore, invoked to explain the effects of HHV-8 on B cells. It may be hypothesized that HHV-8 infection may perturb the homeostasis of B lymphocytes through anti-apoptotic viral proteins, cytokines or other soluble mediators able to affect the proliferation, differentiation, trafficking and, in particular, the lifespan of B cells. These mediators can be produced by lymphocytes, dendritic cells and macrophages in peripheral lymphoid organs, where long-term latency is established. Among other factors, IL-6 of either viral or human origin may represent a good candidate factor, as it is a well defined B cell growth factor [37] centrally involved in KS. It has proven anti-apoptotic effects on B cells [38], [39] and it is expressed by a multitude of cell types upon HHV-8 infection [40], [41]. We ourselves previously demonstrated that dendritic cells from cKS patients produce higher amounts of IL-6 than controls [42]. The lack of increased human-IL-6 levels in our patients and the lack of correlation between circulating IL-6 and B cell changes cannot rule out either the involvement of v-IL-6 or a local effect exerted by IL-6 within lymphoid tissues, namely the spleen.

A disruption of the balance between peripheral B cell subsets has been described in other infectious and non infectious pathological conditions including chronic hepatitis C virus (HCV) infection, human immunodeficiency virus (HIV) infection and systemic sclerosis. The mechanisms proposed to explain the disturbance of peripheral B cell homeostasis in the above conditions rely either on peripheral activation of B cells or to increased serum levels of IL-7 reactive to CD4^+^-T cell lymphopenia [19]–[21], [43]–[46]. Because we did not observe neither B cell activation nor increased plasmatic IL-7 in cKS and HSP patients, we suggest that none of the mechanisms supposed to sustain B cell changes in the above conditions could likely underlie the B cell changes that we observed in our patients. Other factors known to affect B cell maturation, such as B cell activating factor (BAFF), IL-5 and IL-21 [18], [47], [48], were not investigated in this study.

At present, we do not have data on the clinical consequences of the expansion of preimmune B cells in cKS patients, but long-term follow-up studies will be needed to determine whether and how the HHV-8-induced perturbation of B cell homeostatsis may favour the development of HHV-8 associated lymphoproliferative malignancies. The possible relevance of MZ-like B cell expansion observed in our patients to the known MZ involvement that may occur in HHV-8-related lymphoproliferative malignancies [49] needs to be explored. Our observations, together with the recent demonstration that MZ-like B cells are clonally expanded in a subset of patients with chronic HCV infection [50] and support Epstein-Barr virus (EBV) infection in different clinical settings [51], [52], may all provide important cues for further investigation of the mechanisms possibly used by lymphotropic viruses to promote the development of lymphoproliferative disorders.

In conclusion in this study we report, for the first time to our knowledge, that HHV-8 chronic infection promotes a profound perturbation of peripheral B cell homeostasis characterized by expansion of B cells of the preimmune/natural effector compartment, in patients with cKS without clinical signs of humoral immune deficiency. This novel observation may broaden our understanding of the complex interplay of viral and cellular factors leading HHV-8-infected individuals to develop either KS or lymphoproliferative malignancies. Perspectively, long-term follow-up analysis of peripheral B cell changes in cKS patients will greatly help the comprehension of the mechanisms of viral tumorigenesis and possibly enable the development of efficient targeted therapies.

## Materials and Methods

### Patients and controls

Forty-seven cKS patients were included in the study, 39 males and 8 females, mean age 72 years (range 49–95). All patients had histologically confirmed diagnosis of KS, were positive for anti-HHV-8 antibody and negative for HIV. Staging was performed in accordance with our classification that takes into account the prevalent type of lesions, localization, clinical behavior, evolutive pattern, and presence of complications [53], [54]. Patients in systemic chemotherapy were excluded. B cells characterization was performed at a single time point on fresh peripheral blood samples; staging at this time is reported in Table 3. Forty-three age- and sex-matched healthy HHV-8-seronegative subjects were included as controls (HC). Ten age- and sex-matched healthy HHV-8-seropositive (HSP) controls without KS were also enrolled. None of the subjects had suffered from chronic inflammatory, autoimmune and cancer disease other than KS, nor they had clinically evident infections. Peripheral blood samples were obtained by venipuncture and collected into sodium citrate Vacutainer tubes (Becton Dickinson, San Jose, CA, USA).

**Table 3:** Clinical characteristics of cKS patients.

Characteristics		Overall patients	Evolutive pattern	
No. of patients		47		
Age, yr^a^		72.2±1.4		
Sex, no.				
	Male	39		
	Female	8		
KS stage^b^, no.				
			A (slow)	B (rapid)
	I: maculo-nodular	29	15	14
	II: infiltrative	11	4	7
	III: florid	4	1	3
	IV: disseminated	3	0	3
Total			20	27

a Mean ± standard error.

b cKS patients were classified according to our classification that takes into account the prevalent type of lesions, localization, clinical behaviour, evolutive pattern and presence of complications ^(53,54)^.

A indicates slow evolution; B, rapid evolution; rapid denotes an increase in the total number of nodules/plaques or in the total area of plaques in three months following the last examination.

**Ethics statement.** Ethics approval was obtained from the Ethics Committee of the Fondazione Istituto di Ricovero e Cura a Carattere Scientifico Ca' Granda Ospedale Maggiore Policlinico, and written informed consent was provided by study participants.

### Clinical tests

All patients and controls underwent a detailed clinical, haematological and biochemical examination. Plasmatic HHV-8 DNA was quantified in clinical laboratories by HHV-8 Q-PCR Alert Kit (Nanogen Advanced Diagnostics, Milan, Italy), according to the manufacturer's instructions; results were expressed in genome Equivalents (gEq). Anti-HHV-8 specific IgG antibodies were tested in serum samples using a commercial immunofluorescence assay (HHV-8 IgG IFA, Biotrin, Ireland).

### Immunophenotypic analysis of B lymphocytes

Whole peripheral blood (PB) samples were processed within 6 hours after blood withdrawal. 100 µl of PB were washed twice in PBS with 1% bovine serum albumin (BSA) and then incubated in fetal bovine serum (FBS) for 30 min at 37°C. Then, PB samples were stained with various monoclonal antibody (mAb) combinations for 20 min at RT. The following directly conjugated mouse anti-human mAbs were used at optimal concentrations: CD5-allophycocyanin (APC), CD19-peridinin chlorophyll protein (PerCP)-Cyanin (Cy)5.5, CD80-phycoerythrin (PE), CD86-fluorescein isothiocyanate (FITC) (all from BD Biosciences, San Jose, CA, USA); CD20-PE-Cy7 and CD27-APC-Cy7 (from eBiociences, San Diego, CA); CD38-PE (Dako, Glosturp, Denmark); IgD-FITC and IgM-PE (from Serotec, Oxford, UK); κ light chain-FITC and λ light chain-PE (from Miltenyi-Biotec GmbH, Germany); HLA-DR-PE (from Caltag Laboratories, Burlingame, CA). After incubation, erythrocytes were lysed by incubation with ammonium chloride for 10 min at 4°C in the dark. Six-color data acquisition and analysis were performed on a FACSCanto2 flow cytometer (Becton Dickinson) using a FACSDiva software (v6.1) immediately after staining. As isotype controls were not used, those cells that did not express a certain marker were considered as negative control for positive cells [55]. Lymphocytes were gated on forward (FSC) vs side scatter (SSC) plots and B lymphocytes were identified as cells positive for CD19 expression. At least 100,000 lymphocyte-gated cells were routinely collected for each sample. Estimates of the absolute numbers of B cells and their subsets were calculated from the proportion of cells recorded by flow cytometry in the lymphocyte gate multiplied by absolute lymphocyte count measured using a standard hemacytometer.

### Assessment of spontaneous apoptosis of B cell subsets

The sensitivity of B cell subsets to spontaneous apoptosis was evaluated in cultured cells, as described [30], [56]. Isolated PBMCs from 6 cKS patients and 6 HHV-8-seronegative healthy controls were cultured in 24-well plated for 24 h in RPMI 1640 (Euroclone, Wetherby, UK) containing 10% fetal calf serum (Gibco, Invitrogen Co., Carlsbad, CA, USA) and incubated in a 5% CO_2_ incubator at 37°C for 24 hours. Cells were harvested and stained for flow cytometric analysis using an annexin-V APC apoptosis detection kit (BD Pharmingen, San Jose, CA, USA) in combination with anti-CD19-PerCP-Cy5.5, anti-CD27-PE (BD Biosciences) and anti-IgD-FITC (Serotec). Apoptotic cells were identified as annexin-V^+^ cells gated on different B cell subsets.

### Ex vivo Ki67 staining

PBMCs from 6 cKS patients and 6 HHV-8-seronegative healthy controls were stained for flow cytometric analysis with anti-CD19-PerCP-Cy5.5 anti-CD27-PE (BD Biosciences). Samples were then fixed, permeabilised and stained for the cell cycle-associated antigen Ki67 (FITC-labeled B56; BD Biosciences), using the Leucoperm Reagent (AbD Serotec), according to the manufacturer's instructions. Cells that had recently divided were identified as Ki67^+^ cells and provided an estimate of the *in vivo* cell turnover.

### Isolation of human B cells

B lymphocytes were isolated from 7 cKS patients and 6 HSP controls, chosen among subjects with B cell count either over the 75^th^ percentile or under the 25^th^ percentile calculated for each group. Peripheral blood mononuclear cells (PBMCs) were obtained by Ficoll density gradient centrifugation (Ficoll-Hypaque Pharmacia Biotech AB, Sweden). CD19^+^ cells were purified using immunomagnetic selection with mini-MACS cell isolation Kit (Milteny-Biotec) according to the manufacturer's instructions. Total PBMCs, B cells and non-B cells were further processed for virological analyses.

### Assessment of HHV-8 infection in isolated cells

Isolated PBMCs, B cells and non-B cells from 7 cKS patients and 6 HSP controls were tested for the presence and load of HHV-8 DNA sequences, as previously described [8], [57]. Briefly, DNA was extracted from at least 2×10^5^ cells, and HHV-8 viral load was measured by quantitative real-time polymerase chain reaction (PCR), and results were reported as gEq/10^5^ cells.

### IL-6 and IL-7 measurement in plasma samples

Plasma samples were obtained under sterile conditions from single donors. The amounts of plasmatic IL-6 and IL-7 were determined by ELISA kits from R&D Systems (Minneapolis, MN). All individual steps were performed according to the manufacturer's instructions.

### Statistical analysis

Data are presented as mean ± SE. Comparisons of samples to establish the statistical significance of difference were determined by the Student *t* test for independent samples. The Mann-Whitney *U* test was also used when indicated. All statistical analyses assumed a 2-sided significance level of 0.05. Data analyses were performed with Openstat3 software.
